# Rare c.302C>T *TTR* Variant Associated with Transthyretin Amyloidosis

**DOI:** 10.3390/medicina60020237

**Published:** 2024-01-30

**Authors:** Dovilė Žebrauskienė, Eglė Sadauskienė, Rūta Masiulienė, Sigita Aidietienė, Agnė Šiaudinienė, Valdas Pečeliūnas, Gabrielė Žukauskaitė, Edvardas Žurauskas, Nomeda Valevičienė, Jūratė Barysienė, Eglė Preikšaitienė

**Affiliations:** 1 Department of Human and Medical Genetics, Institute of Biomedical Sciences, Faculty of Medicine, Vilnius University, LT-03101 Vilnius, Lithuania; 2 Clinic of Cardiac and Vascular Diseases, Institute of Clinical Medicine, Faculty of Medicine, Vilnius University, LT-03101 Vilnius, Lithuania; 3 Faculty of Medicine, Vilnius University, LT-03101 Vilnius, Lithuania; 4 Center of Haematology, Oncology and Transfusion Medicine, Vilnius University Hospital Santaros Klinikos, LT-08661 Vilnius, Lithuania; 5 Clinic of Internal Medicine, Family Medicine and Oncology, Institute of Clinical Medicine, Faculty of Medicine, Vilnius University, LT-03101 Vilnius, Lithuania; 6 National Center of Pathology, Vilnius University Hospital Santaros Klinikos, LT-08406 Vilnius, Lithuania; 7 Department of Radiology, Nuclear Medicine and Medical Physics, Institute of Biomedical Sciences, Faculty of Medicine, Vilnius University, LT-03101 Vilnius, Lithuania

**Keywords:** transthyretin, cardiac amyloidosis, *TTR* gene, c.302C>T

## Abstract

*Background and Objectives:* Hereditary transthyretin amyloidosis (ATTRv) is a rare disease caused by pathogenic variants in the transthyretin (*TTR*) gene. More than 140 different disease-causing variants in *TTR* have been reported. Only a few individuals with a rare *TTR* variant, c.302C>T, p.(Ala101Val) (historically known as p.(Ala81Val)), primarily associated with cardiac ATTRv, have been described. Therefore, our aim was to analyze the clinical characteristics of individuals with the identified c.302C>T *TTR* variant at our center. *Materials and Methods:* We analyzed data from individuals with ATTRv who were diagnosed and treated at Vilnius University Hospital Santaros Klinikos. ATTRv was confirmed by negative hematological analysis for monoclonal protein, positive tissue biopsy or bone scintigraphy and a pathogenic *TTR* variant. *Results:* During 2018-2021, the *TTR* NM_000371.3:c.302C>T, NP_000362.1:p.(Ala101Val) variant was found in one individual in a homozygous state and in three individuals in a heterozygous state. The age of onset of symptoms ranged from 44 to 74 years. The earliest onset of symptoms was in the individual with the homozygous variant. A history of carpal tunnel syndrome was identified in two individuals. On ECG, three individuals had low QRS voltage in limb leads. All individuals had elevated NT-proBNP and hsTroponine I levels on baseline laboratory tests and concentric left ventricular hypertrophy on transthoracic echocardiography. The individual with the homozygous c.302C>T *TTR* variant had the most pronounced polyneuropathy with tetraparesis. Other patients with the heterozygous variant had more significant amyloid cardiomyopathy. When screening family members, the c.302C>T *TTR* variant was identified in two phenotypically negative relatives at the ages of 33 and 47 years. *Conclusions:* c.302C>T is a rare *TTR* variant associated with ATTRv cardiomyopathy. The homozygous state of this variant was not reported before, and is associated with earlier disease onset and neurological involvement compared to the heterozygote state.

## 1. Introduction

Hereditary transthyretin amyloidosis (ATTRv) is a rare autosomal dominant disease caused by pathogenic variants in the transthyretin (*TTR*) gene on chromosome 18 [1]. Amyloidogenic pathogenic variants destabilize the native TTR and promote the dissociation of the tetramer into partially dispersed forms, which self-assemble into amyloid fibrils and form amyloid deposits in the extracellular space of various tissues and organs [1]. Phenotypes associated with ATTRv are cardiac amyloidosis, amyloid neuropathy and leptomeningeal amyloidosis/cerebral amyloid angiopathy [2].

More than 140 *TTR* disease-causing variants have been described in ATTRv [3]. The prevalence of different *TTR* variants varies according to geographical region and ethnicity. Few genotype–phenotype correlations have been observed [4]. The most common pathogenic *TTR* variant associated with cardiac amyloidosis is c.424G>A, p.(Val142Ile), with a prevalence of 3.4% among African-Americans [5]. Other frequent *TTR* pathogenic variants associated with the cardiac phenotype are c.391C>A, p.(Leu131Met) and c.262A>T, p.(Ile88Leu) [6]. The most common pathogenic *TTR* variant causing ATTRv polyneuropathy is c.148G>A, p.(Val50Met) (previously known as Val30Met), which is endemic in some regions of Portugal, Sweden and Japan [7,8]. The representative genotype for ATTRv leptomeningeal amyloidosis is c.113A>G, p.(Asp38Gly), first described in a Hungarian family [9].

A rare *TTR* variant, c.302C>T, p.(Ala101Val) (historically known as p.(Ala81Val)), has been described in few individuals with cardiac ATTRv [10,11,12,13]. The aim of this study was to further delineate the cardiac phenotype resulting from the *TTR* c.302C>T variant in the heterozygous or homozygous state, detected in four affected individuals at our center.

## 2. Materials and Methods

We reviewed the data of individuals diagnosed with ATTRv at Vilnius University Hospital Santaros Klinikos during the years 2018–2021. The diagnosis of ATTRv was confirmed by negative hematological analysis, positive histological or non-invasive (^99m^Tc-labeled pyrophosphate (^99m^Tc-PYP) scintigraphy) analysis for ATTRv and the identified pathogenic/likely pathogenic variant in the *TTR* gene. Individuals with the confirmed *TTR* c.302C>T variant were included for the further evaluation. This study was approved by the Vilnius Regional Biomedical Research Ethics Committee. Written informed consent was obtained from all probands and their examined relatives. 

### 2.1. Clinical Evaluation

We present four unrelated families with four affected individuals who carry a *TTR* c.302C>T variant.

**Figure 1:**
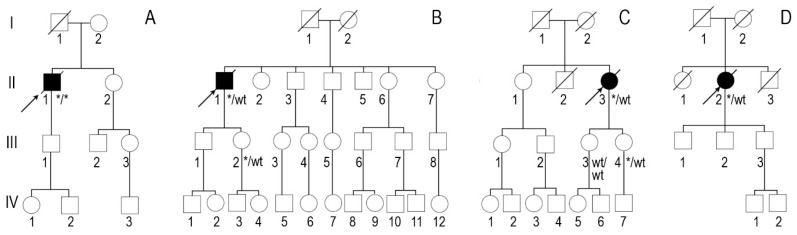
Genealogies: (**A**) family 1, (**B**) family 2, (**C**) family 3 and (**D**) family 4. Arrows show probands. Asterisks show familial variant NM_000371.3:c.302C>T, NP_000362.1:p.(Ala101Val).

**Figure 2:**
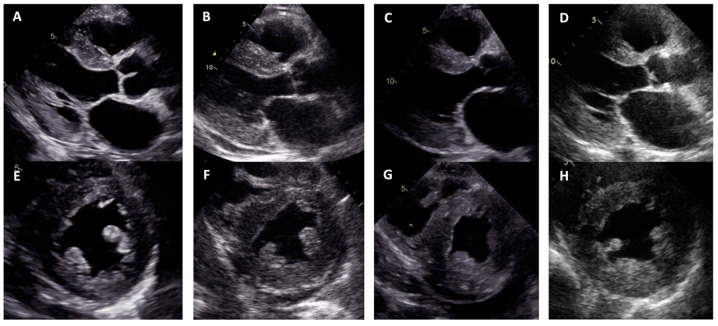
Concentric left ventricular hypertrophy in transthoracic echocardiography views: (**A**–**D**) parasternal long axis; (**E**–**H**) parasternal short axis; (**A**,**E**) proband, family 1; (**B**,**F**) proband, family 2; (**C**,**G**) proband, family 3; (**D**,**H**) proband, family 4.

**Figure 3:**
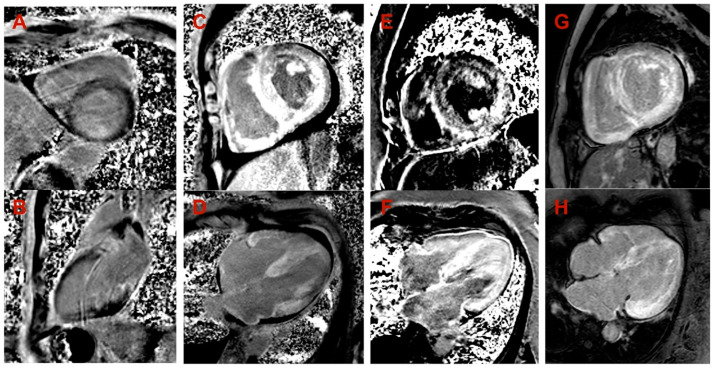
Late gadolinium enhancement (LGE) images in cardiac magnetic resonance. (**A**,**B**) Proband, family 1: (**A**) midmyocardial LGE in the septum (short axis view); (**B**) midmyocardial LGE in inferior wall (2-chamber view). (**C**,**D**) Proband, family 2: diffuse transmural (predominantly subendocardial) LGE in LV and RV, LV and RV hypertrophy, small amount of pericardial fluid (short axis view in (**C**), 4-chamber view in (**D**)). (**E**,**F**) Proband, family 3: diffuse midmyocardial LGE in LV and RV, LV and RV hypertrophy (short axis view in (**E**), 4-chamber view in (**F**)). (**G**,**H**) Proband, family 4: diffuse subendocardial LGE in LV and RV, LV and RV hypertrophy (short axis view in (**G**), 4-chamber view in (**H**)).

**Figure 4:**
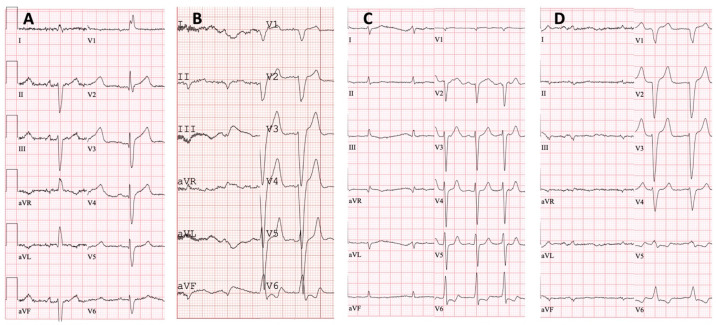
Twelve-lead electrocardiograms: (**A**) proband, family 1—sinus rhythm, right bundle branch block and left anterior fascicular block; (**B**) proband, family 2—atrial fibrillation, left bundle branch block and low QRS voltage in the limb leads; (**C**) proband, family 3—atrial fibrillation and low QRS voltage in the limb leads; (**D**) proband, family 4—atrial fibrillation, left bundle branch block and low QRS voltage in the limb leads.

**Figure 5:**
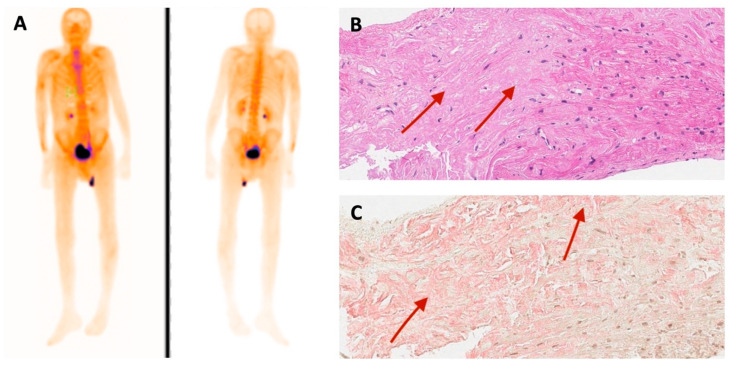
(**A**) ^99m^Tc-PYP bone scintigraphy and (**B**) histological findings of endomyocardial biopsy of family 1 proband. Hematoxylin and eosin stain. Red arrows show amorphous eosinophilic deposits in myocardium (amyloid). (**C**) Congo red stain. Red arrows indicate amorphous amyloid deposits (in pink).

Family 1. At the age of 44 years, the male proband noticed numbness in the toes of his right foot, which quickly spread to both limbs (Table 1, Proband No. 1; Figure 1A, II-1). The numbness progressed over time, with paresthesia in the arms, legs, and trunk up to the navel. The proband was examined by a neurologist and diagnosed with sensomotor polyneuropathy of the upper and lower extremities and pronounced tetraparesis at the age of 45 years. He also suffered from diarrhea, impaired pelvic organ function, weight loss and swelling. Cardiac involvement was diagnosed 4 years later. The first cardiological evaluation showed normal voltages on electrocardiogram (ECG), transthoracic echocardiography (TTE) revealed concentric left ventricular (LV) hypertrophy (max. 13 mm) (Figure 2A,E) with normal ejection fraction (EF) (LVEF 67%) and troponin I and NT-pro-BNP were elevated. Cardiac magnetic resonance imaging (MRI) showed asymmetric LV hypertrophy (predominantly in the interventricular septum) with suspected hypertrophic cardiomyopathy. Midmyocardial gadolinium accumulation of up to 50% in the inferior LV wall and focal fibrotic lesions in the hypertrophied part of the basal interventricular septum were observed (Figure 3A,B). Later ECG revealed a right bundle branch block and a left anterior fascicular block (Figure 4A). Coronary computed tomography angiography showed no abnormalities in the coronary arteries. Hematological analysis for AL amyloidosis was negative. Adipose tissue, duodenal, colon biopsy and bone marrow trepanobiopsy revealed amyloid deposition with non-specific transthyretin immunohistochemistry reaction. However, ^99m^Tc-PYP bone scintigraphy showed no myocardial uptake (Perugini grade 0) (Figure 5A). Subsequently, endomyocardial biopsy was performed for differential diagnosis. Amyloid deposits were found (Figure 5B,C), but immunohistochemistry showed a likely non-specific reaction for the transthyretin. Mass spectrometry was not available. At the age of 52 years, the proband died due to complications of pneumonia.

According to the family history (Figure 1A), the father (I-1) of the proband (II-1) was diagnosed with Parkinson’s disease and died at the age of 68. The mother had an unspecified heart disease. The sister (II-2), aged 46, underwent surgery for carpal tunnel syndrome on both sides. An aunt on the mother’s side had an unspecified heart disease. Both parents of the proband were of Polish descent. Family members were not available for genetic testing. 

Family 2. At the age of 74 years, the male proband underwent cardiological examination due to dyspnea and chest pain that had persisted for a few years (Table 1, Proband No. 2, Figure 1B, II-1). TTE showed severely reduced systolic function and concentric hypertrophy of both ventricles (Figure 2B,F). Cardiac MRI revealed a restrictive cardiomyopathy phenotype with reduced LVEF to 45% and significant symmetric LV hypertrophy. Diffuse myocardial infiltration with gadolinium (predominantly subendocardial) was observed in the LV and right ventricular (RV) myocardium (Figure 3B,F). There was significant elevation of NT-proBNP and a mild increase in troponine I. Serum-free light chains, serum, and urine immunofixation were negative. Histological examination of an abdominal fat aspirate revealed amyloid in the adipose tissue (transthyretin amyloid deposition). There was no history of carpal tunnel syndrome. The proband had permanent atrial fibrillation and left bundle branch block on ECG (Figure 4B), stage III chronic kidney disease and chronic superficial gastritis. Two years later, at the age of 79 years, the proband died due to colon adenocarcinoma.

According to the genealogy (Figure 1B), the proband’s (II-1) brother (II-3) had an unspecified heart disease. The familial *TTR* variant was detected in the propband’s phenotypically negative 47-year-old daughter (III-2). She underwent TTE and cardiac MRI with no signs of cardiac amyloidosis. Other family members were not available for genetic testing.

Family 3. The proband, a 50-year-old female, presented to an outpatient cardiology clinic because of worsening dyspnea on exertion and lower-extremity edema that had persisted for 12 months (Table 1, Proband No. 3, Figure 1C, II-3). Since the age of 36, she had been diagnosed and treated for primary arterial hypertension and heart failure NYHA class II. She was also diagnosed with inflammatory polyarthropathy and was treated with steroids and methotrexate. At the outpatient clinic, TTE revealed concentric LV hypertrophy with maximal wall thickness of 14 mm, preserved ejection fraction (LVEF 55%), restrictive LV filling pattern, biatrial enlargement and signs of pulmonary hypertension (Figure 2C,G). A chest computed tomography with angiography was performed for suspected pulmonary thromboembolism, but no thrombi were detected in the central and segmental branches of the pulmonary artery. Additionally, lung ventilation-perfusion scintigraphy was performed, which revealed a small branch pulmonary embolism in the left lung. Anticoagulation therapy was initiated. The proband was referred to a hematologist for suspected thrombophilia. Elevated factor VIII activity was found and genetic analysis showed the c.126A>C *MTHFR* gene variant in the heterozygous state. However, the heterozygous variant is not associated with hyperhomocysteinemia or increased risk of venous or arterial thrombosis. Coronary angiography was performed to exclude coronary artery disease and showed significant stenosis (80%) at the proximal segment of the right coronary artery. Therefore, percutaneous coronary intervention with stent implantation followed. Subsequently, cardiac MRI was performed, which revealed symmetric LV hypertrophy with slightly reduced ejection fraction (LVEF 50%) and diffuse midmyocardial late gadolinium enhancement (LGE) in the LV and RV (Figure 3E,F). Accordingly, infiltrative cardiomyopathy with the highest likelihood of amyloidosis was considered. Three years later, bilateral carpal tunnel syndrome was surgically treated. Finally, biopsy of adipose tissue and myocardium was performed, and histological analysis confirmed transthyretin cardiac amyloidosis. Later, ECG revealed persistent atrial fibrillation (Figure 4C). Cardioversion was withheld because repeated transesophageal echocardiography showed persistent thrombus in the left atrial appendage despite adequate anticoagulation with vitamin K antagonist. During the following years, repeated pleural drainage was performed due to pleural effusions; therefore, a long-term pleural catheter was implanted. The proband died at the age of 60 due to heart failure decompensation. 

The family history revealed that the proband’s mother (I-2) and maternal grandmother had unexplained heart disease (Figure 1C). The familial *TTR* variant was identified in one phenotypically negative daughter at the age of 33 years (III-4). She underwent TTE and cardiac MRI, which showed with no signs of cardiac amyloidosis. Other family members were not available for genetic testing.

Family 4. The proband, a 72-year-old female, was admitted to the hospital for the first time after suffering a stroke (Table 1, Proband No. 4, Figure 1D, II-2). Urgently, thrombectomy was performed and complete symptom regression was observed. During hospitalization, ECG showed tachycardia, atrial fibrillation, left bundle branch block and low QRS voltage in the limb leads (Figure 4D). The proband denied palpitations but complained of worsening dyspnea on exertion and lower-extremity edema that had been noted for several years. Sinus rhythm was restored by electrical cardioversion. She denied any other chronic illnesses and medication use. TTE showed concentric LV hypertrophy with maximal wall thickness of 19 mm, reduced ejection fraction (LVEF 40%) and restrictive LV filling pattern (Figure 2D,H). Troponin I and NT-pro BNP were significantly elevated. Adipose tissue biopsy showed amyloid deposition. ^99m^Tc-PYP bone scintigraphy revealed grade 3 myocardial uptake suggestive of ATTRv amyloidosis. Coronary angiography showed no abnormalities in the coronary arteries. Cardiac MRI revealed concentric LV hypertrophy with maximal wall thickness of 20 mm, reduced LVEF (44%) and diffuse subendocardial LGE in the LV and RV (Figure 3G,H). Carpal tunnel syndrome was confirmed by a neurologist after a comprehensive evaluation. The woman did not consistently comply with prescribed treatment, atrial fibrillation returned, and heart failure (dyspnea and edema) progressed, thus she was hospitalized several times. After 1 year of follow-up, the proband died due to heart failure decompensation. 

According to the genealogy (Figure 1D), the proband’s mother (I-2) and brother (II-3) died from stroke at the ages of 87 and 77, respectively. Her father (I-1) died at the age of 65 after surgery for suspected thrombosis, and her sister (II-1) died at the age of 60 from colorectal cancer. Family members were not available for genetic testing.

### 2.2. Genetic Evaluation

#### 2.2.1. DNA Extraction

DNA was isolated from the peripheral blood using the phenol-chloroform-isoamyl alcohol method.

#### 2.2.2. Next Generation Sequencing

For the probands from families 1 and 2, next-generation sequencing (NGS) analysis of genomic DNA was performed using TruSightOne Sequencing panels (Illumina Inc., San Diego, CA, USA). For the probands from families 3 and 4, NGS of genomic DNA was performed using Human Core Exome Kits (Twist Bioscience, South San Francisco, California, USA) as previously described [14]. *APOA1*, *APOA2*, *APOC2*, *APOC3*, *B2M*, *CST3*, *FGA*, *GSN*, *NLRP3 LYZ* and *TTR* genes, associated with amyloidosis, were analyzed. Variants were classified following the guidelines of the American College of Medical Genetics and Genomics (ACMG) [15]. Only variants that passed quality and coverage filters and showed >99.9% detection reliability were analyzed.

#### 2.2.3. Sanger Sequencing

For the following segregation analysis, Sanger sequencing was performed using gDNA samples of the proband’s sister and daughter in family 2 and the proband’s two daughters in family 3. Polymerase chain reaction (PCR) of gDNA sequence flanking the familial variant of the *TTR* gene was performed using specific primers designed with the Primer Blast tool (available upon request). PCR was performed using Phusion High-Fidelity PCR Master Mix (Thermo Fisher Scientific, Waltham, Massachusetts, USA). PCR products were fractioned by 1.5% agarose gel (TopVision, Thermo Fisher Scientific, USA) electrophoresis and visualized under ultraviolet light. The PCR products were sequenced with BigDye^®^ Terminator v3.1 Cycle Sequencing Kits (Thermo Fisher Scientific, USA) and ABI 3130xL Genetic Analyser (Thermo Fisher Scientific, USA). The resulting sequences were aligned with the reference sequence of *TTR* (NCBI: NM_000371.3).

#### 2.2.4. Real-Time PCR Analysis

Real-time PCR analysis was performed for the family 1 proband to exclude a hemizygosity due to deletion. A pair of primers specific to the deletion region of exon 3 in the *TTR* (MIM#176300) gene was designed for this purpose (forward primer: 5′-TGTTTCCTCCATGCGTAACT-3′; reverse primer: 5′-AAACCAAAACAACCCTCGAAG-3′). *SERPINB13* (MIM#604445) gene primers (forward primer: 5′-CCAGGTGTTTCACTCTGAAAAAGA-3′; reverse primer: 5′-CCCAGAGACCCCACACCTAA-3′) were used to amplify the target in exon 3, which was considered as endogenous control outside the deletion region in the same chromosome 18. Additionally, *RNASE2* (MIM#131410) gene primers (forward primer: 5′-TGCGAAACTGCGTGGACATT-3′; reverse primer: 5′-ATGCGGAAGCCCATTTCCAT-3′) were used to amplify a region of interest outside chromosome 18, serving as a second endogenous control and normalizer gene. Primers were created using the Primer3 program [16]. Control samples from both sexes without the clinical phenotype and a negative reaction control (water) were included in the analysis.

One real-time PCR reaction contained 1.5 pmol of each primer and SYBR Green PCR Mix (Applied Biosystems, Waltham, Massachusetts, USA). Each reaction was replicated 3 times using 10 μg/mL of genomic DNA (1.2 μL) in a 15 μL total reaction volume. Real-time PCR was conducted using the ABI Prism 7900HT sequence detection system and SDS software v2.3 (Applied Biosystems) according to the following conditions: initiation at 50 °C for 2 min, initial denaturation at 95 °C for 2 min, followed by 40 cycles of denaturation at 95 °C for 15 s with annealing and extension at 60 °C for 1 min. Finally, an additional dissociation stage was added at 95 °C for 15 s and at 60 °C for 15 s. The obtained results were interpreted in a relative quantification approach using the Comparative Ct (ΔΔCt) method normalizing to endogenous control (*RNASE2* gene).

## 3. Results

Next-generation sequencing of the family 1 proband revealed a likely pathogenic homozygous variant NM_000371.3:c.302C>T, NP_000362.1:p.(Ala101Val), rs1555631417 in the *TTR* gene. As parental testing was not available, real-time PCR analysis was performed, and no heterozygous deletion of exon 3 of the *TTR* gene was detected. To our knowledge, this variant in the homozygous state has not been described in the literature before. For the three other non-related probands (families 2, 3 and 4), the same variant was found in the heterozygous state. The familial variant was also detected in the proband’s daughter in both family 2 and family 3.

This missense variant is located in coding exon 3 of the *TTR* [17]. It is not present in large population databases (GnomAD—0). The variant is located in a hot-spot of pathogenic missense variants in *TTR*. ClinVar classifies this variant as of likely pathogenic/uncertain significance. This heterozygous variant has been previously reported in the literature in patients with cardiac amyloidosis [11,13,18].

## 4. Discussion

We report four unrelated Lithuanian individuals with rare *TTR* gene variant c.302C>T (one homozygous and three heterozygous) at a single center and present detailed evaluation of their cardiac phenotypes. Only a few individuals with the c.302C>T *TTR* variant have been reported before. This variant has most commonly been found in individuals of Polish and Russian descent and is mostly associated with cardiac ATTRv [2,10,11,12,13].

The c.302C>T variant was first described in 2007 as a p.Ala81Val amino acid change. At the time, the variant was associated with heart involvement and was found in the United Kingdom [18]. In studies by D. Rowczenio et al., the c.302C>T (p.Ala101Val) *TTR* variant was identified in three Polish and Russian individuals with the cardiac phenotype [11,12]. The individual with the homozygous *TTR* c.302C>T variant in our study was also of Polish descent. In a Polish study of 10 patients diagnosed with hereditary ATTRv, only one patient was identified as having the c.302C>T *TTR* variant. He was a 67-year-old male with a severely hypertrophied LV and RV (max. thickness of interventricular septum was 2.7 cm), reduced LV systolic function (LVEF 35%) and progressive HF (NYHA functional class II/III). This individual also had atrial fibrillation, bilateral carpal tunnel syndrome, orthostatic hypotension and mild polyneuropathy. There was no positive family history. The proband was diagnosed with cardiac ATTRv via mass spectrometry as the hematological analysis showed monoclonal gammopathy of unknown significance and tissue biopsy revealed only microdeposits of amyloid. He died 4 years later at the age of 71 due to stroke [10]. Only one Chinese male patient with c.302C>T *TTR* variant was reported. ATTRv amyloidosis started with bilateral carpal tunnel syndrome at 58 years of age; seven years later, lumbar spondylosis developed. The disease then progressed rapidly, and within a year, cardiomyopathy and orthostatic hypotension developed. TTR amyloid deposits were identified by mass spectrometry from a sural nerve biopsy specimen. His mother had a history of neuropathy. The patient died at the age of 67 due to pneumonia [13]. In our study, the proband with the homozygous *TTR* c.302C>T variant had the most pronounced polyneuropathy with tetraparesis. Cardiac involvement was diagnosed 4 years later. Other heterozygous patients had more significant amyloid cardiomyopathy.

Homozygous *TTR* gene variants are rare. The homozygous variant *TTR* c.302C>T has not been previously described in the literature to our knowledge. However, other homozygous variants of the *TTR* gene have been reported [19,20,21,22,23,24]. The mean age at cardiac ATTRv diagnosis was 10 years younger for individuals with the homozygous Val122Ile variant than those with the heterozygous variant (64 ± 6 years versus 72 ± 8 years) [21]. As in our report, the proband with the homozygous variant had the earliest onset of symptoms at 44 years, approximately 6 years earlier than the family 3 proband with a heterozygous variant. Homozygous patients tend to have higher misfolded (variant) TTR serum levels than heterozygous patients. In a study by Holmgren G. et al., the level of variant TTR in two homozygous individuals with familial amyloidotic polyneuropathy was twice as high as that in other heterozygous individuals with Val30Met variants [25]. In an animal model, mice with homozygous human TTR (Val30Met) produced twice as much human TTR mRNA and protein in the liver as heterozygous mice with mouse/human TTR [26]. Therefore, homozygous patients may have a more severe form of the disease and develop symptoms earlier than heterozygous individuals [27].

In a proband with a homozygous *TTR* c.302C>T variant, ^99m^Tc-PYP bone scintigraphy was negative, though amyloid deposits were detected in endomyocardial biopsy. Immunohistochemistry could not specify the amyloid type as it showed a non-specific reaction to transthyretin, and mass spectrometry was not available at our center. In the absence of monoclonal protein in serum and urine, the specificity and positive predictive value of bone scintigraphy are almost 100% [28]. Nevertheless, there are some situations that can lead to negative cardiac uptake: very early disease, rib fracture, recent myocardial infarction, premature or delayed acquisition and some *TTR* variants with usually neurological involvement [29]. Bone scintigraphy can produce false negative results in patients with type B amyloid fibres that are found in early-onset Val30Met and Tyr114Cys variants [30]. Type B fibres consist of full-length TTR, and type A fibres consist of a mixture of full-length TTR and C-terminal fragments [31]. Individuals with type B amyloid fibrils tend to have early onset of disease and less clinical cardiac involvement than type A patients [32]. In patients with the *TTR* Phe64Leu variant, the sensitivity of bone scintigraphy with ^99m^Tc–diphosphonate or ^99m^Tc–hydroxyl-methylene diphosphonate was reported to be extremely low (10.5%) in detecting cardiac ATTRv [33]. Also, low sensitivity was reported in Ser9Tyr *TTR* variants [29]. If the amyloid type remains unclear, mass spectrometry is recommended to support the diagnosis [29,34].

## 5. Conclusions

The rare *TTR* c.302C>T variant is associated with cardiac ATTRv. This is the first report of a homozygous c.302C>T *TTR* variant. The earlier disease onset and neurological involvement in the homozygous state suggest that, like other TTR gene variants, the homozygous variant of this gene may be associated with more severe ATTRv. More detailed studies are needed to clarify the differences between patients with homozygous *TTR* variants and those with heterozygous variants.

## Figures and Tables

**Table 1:** Baseline clinical findings and outcomes of the individuals with likely pathogenic *TTR* gene variant c.302C>T.

Proband No.	1	2	3	4
Zygosity of NM_000371.3:c.302C>T, NP_000362.1:p.(Ala101Val) variant in *TTR*	Homozygous	Heterozygous	Heterozygous	Heterozygous
Sex	Male	Male	Female	Female
Age of onset of symptoms (years)	44	74	50	72
Age at diagnosis (years)	49	77	57	74
NYHA class of heart failure	III	III	III	III
Positive family history	Mother at an older age and aunt on mother‘s side had heart disease	Brother had heart disease	Mother had heart disease, grandmother on mother‘s side had sudden death	Mother and brother died of stroke at an older age
Low QRS voltage	-	+	+	+
Pseudoinfarct pattern on ECG	-	-	-	-
Conduction disturbances	RBBB, LAFB	LBBB	-	LBBB
Atrial fibrillation	-	+	+	-
LV hypertrophy	Concentric	Concentric	Concentric	Concentric
Maximal wall thickness (mm)	13	21	14	19
LVEF (%)	67	10	55	40
Restrictive LV filling pattern	-	+	+	+
Increased RV wall thickness	+	+	-	+
Pericardial effusion	-	+	-	-
Cardiac MRI LV hypertrophy	Asymmetric (predominantly in transventricular septum)	Symmetric	Symmetric	Symmetric
Cardiac MRI maximal wall thickness (mm)	16	19	14	20
Cardiac MRI LVEF (%)	77	45	50	44
Cardiac MRI LGE	Midmyocardial LGE in LV septum and inferior wall	Diffuse subendocardial LGE in LV and RV	Diffuse midmyocardial LGE in LV and RV	Diffuse subendocardial LGE in LV and RV
^99m^Tc-PYP bone scintigraphy	Grade 0	-	-	Grade 3
Histological confirmation	Amyloid deposits, likely non-specific reaction to transthyretin on immunohistochemistry in bone marrow trepanobiopsy and endomyocardial biopsy	TTR amyloid deposition in adipose tissue biopsy	TTR amyloid deposition in endomyocardial biopsy	-
NT-proBNP (pg/mL) *	474	11401	3368	2471
Troponin I (ng/L) *	111	95	65	45
Polyneuropathy	+	-	-	+
Chronic kidney disease	-	+	+	-
Gastrointestinal manifestation	+	+	-	-
Carpal tunnel syndrome	-	-	+	+
Biceps tendon rupture	-	NA	-	-
Follow-up after diagnosis (years)	3	2	3	1
Outcome	Death at age 52 due to pneumonia complications	Death at age 79 due to colon adenocarcinoma	Death at age 60 due to heart failure decompensation	Death at age 75 due to heart failure decompensation
Family segregation analysis	NA	Variant identified in phenotypically negative 47-year-old daughter	Variant identified in phenotypically negative 33-year-old daughter	NA

* At first evaluation. ECG—electrocardiogram; LAFB—left anterior fascicular block; LBBB—left bundle branch block; LGE—late gadolinium enhancement; LV—left ventricle; LVEF—left ventricle ejection fraction; MRI—magnetic resonance imaging; NA—not applicable; NYHA—New York Heart Association; RBBB—right bundle branch block; RV—right ventricle; TTR—tranthyretin; ^99m^Tc-PYP—^99m^Tc-labeled pyrophosphate.

## Data Availability

The main data generated and analyzed during this study are included in this article. Any additional information is available from the authors upon request.

## References

[1] Manganelli F., Fabrizi G.M., Luigetti M., Mandich P., Mazzeo A., Pareyson D. (2022). Hereditary Transthyretin Amyloidosis Overview. Neurol. Sci. Off. J. Ital. Neurol. Soc. Ital. Soc. Clin. Neurophysiol..

[2] Sekijima Y., Adam M.P., Ardinger H.H., Pagon R.A., Wallace S.E., Bean L.J., Mirzaa G., Amemiya A. (1993). Hereditary Transthyretin Amyloidosis. GeneReviews^®^.

[3] Mutations in Hereditary Amyloidosis. http://www.amyloidosismutations.com/.

[4] Maurer M.S., Bokhari S., Damy T., Dorbala S., Drachman B.M., Fontana M., Grogan M., Kristen A.V., Lousada I., Nativi-Nicolau J. (2019). Expert Consensus Recommendations for the Suspicion and Diagnosis of Cardiac ATTR Amyloidosis. Circ. Heart Fail..

[5] Jacobson D.R., Alexander A.A., Tagoe C., Buxbaum J.N. (2015). Prevalence of the Amyloidogenic Transthyretin (TTR) V122I Allele in 14 333 African-Americans. Amyloid Int. J. Exp. Clin. Investig. Off. J. Int. Soc. Amyloidosis.

[6] Damy T., Kristen A.V., Suhr O.B., Maurer M.S., Planté-Bordeneuve V., Yu C.-R., Ong M.-L., Coelho T., Rapezzi C., THAOS Investigators (2019). Transthyretin Cardiac Amyloidosis in Continental Western Europe: An Insight through the Transthyretin Amyloidosis Outcomes Survey (THAOS). Eur. Heart J..

[7] Koike H., Misu K., Ikeda S., Ando Y., Nakazato M., Ando E., Yamamoto M., Hattori N., Sobue G. (2002). Study Group for Hereditary Neuropathy in Japan Type I (Transthyretin Met30) Familial Amyloid Polyneuropathy in Japan: Early- vs Late-Onset Form. Arch. Neurol..

[8] Ruberg F.L., Grogan M., Hanna M., Kelly J.W., Maurer M.S. (2019). Transthyretin Amyloid Cardiomyopathy: JACC State-of-the-Art Review. J. Am. Coll. Cardiol..

[9] Vidal R., Garzuly F., Budka H., Lalowski M., Linke R.P., Brittig F., Frangione B., Wisniewski T. (1996). Meningocerebrovascular Amyloidosis Associated with a Novel Transthyretin Mis-Sense Mutation at Codon 18 (TTRD 18G). Am. J. Pathol..

[10] Gawor M., Holcman K., Franaszczyk M., Lipowska M., Michałek P., Teresińska A., Bilińska Z.T., Rubiś P., Kostkiewicz M., Szot W. (2020). Spectrum of Transthyretin Gene Mutations and Clinical Characteristics of Polish Patients with Cardiac Transthyretin Amyloidosis. Cardiol. J..

[11] Rowczenio D.M., Noor I., Gillmore J.D., Lachmann H.J., Whelan C., Hawkins P.N., Obici L., Westermark P., Grateau G., Wechalekar A.D. (2014). Online Registry for Mutations in Hereditary Amyloidosis Including Nomenclature Recommendations. Hum. Mutat..

[12] Rowczenio D., Quarta C.C., Fontana M., Whelan C.J., Martinez-Naharro A., Trojer H., Baginska A., Ferguson S.M., Gilbertson J., Rezk T. (2019). Analysis of the TTR Gene in the Investigation of Amyloidosis: A 25-Year Single UK Center Experience. Hum. Mutat..

[13] Chen Z., Koh J.S., Saini M., Tay K.S.S., Jayne Tan Y., Chai J.Y.H., Fam S.R., Juraidah A.R., Lim P.K., Ng A.S.L. (2021). Hereditary Transthyretin Amyloidosis-Clinical and Genetic Characteristics of a Multiracial South-East Asian Cohort in Singapore. J. Neuromuscul. Dis..

[14] Grigaitė J., Šiaurytė K., Audronytė E., Preikšaitienė E., Burnytė B., Pranckevičienė E., Ekkert A., Utkus A., Jatužis D. (2021). Novel In-Frame Deletion in HTRA1 Gene, Responsible for Stroke at a Young Age and Dementia-A Case Study. Genes.

[15] Richards S., Aziz N., Bale S., Bick D., Das S., Gastier-Foster J., Grody W.W., Hegde M., Lyon E., Spector E. (2015). Standards and Guidelines for the Interpretation of Sequence Variants: A Joint Consensus Recommendation of the American College of Medical Genetics and Genomics and the Association for Molecular Pathology. Genet. Med. Off. J. Am. Coll. Med. Genet..

[16] Kõressaar T., Lepamets M., Kaplinski L., Raime K., Andreson R., Remm M. (2018). Primer3_masker: Integrating Masking of Template Sequence with Primer Design Software. Bioinforma. Oxf. Engl..

[17] VCV000495842.10—ClinVar—NCBI. https://www.ncbi.nlm.nih.gov/clinvar/variation/495842/?oq=TTR[gene]+AND+c.302C%3ET[varname]+&m=NM_000371.4(TTR):c.302C%3ET%20(p.Ala101Val).

[18] Benson M.D., Kincaid J.C. (2007). The Molecular Biology and Clinical Features of Amyloid Neuropathy. Muscle Nerve.

[19] Munar-Qués M., López Domínguez J.M., Viader-Farré C., Moreira P., Saraiva M.J. (2001). Two Spanish Sibs with Familial Amyloidotic Polyneuropathy Homozygous for the V30M-TTR Gene. Amyloid Int. J. Exp. Clin. Investig. Off. J. Int. Soc. Amyloidosis.

[20] Jacob E.K., Edwards W.D., Zucker M., D’Cruz C., Seshan S.V., Crow F.W., Highsmith W.E. (2007). Homozygous Transthyretin Mutation in an African American Male. J. Mol. Diagn. JMD.

[21] Reddi H.V., Jenkins S., Theis J., Thomas B.C., Connors L.H., Van Rhee F., Highsmith W.E. (2014). Homozygosity for the V122I Mutation in Transthyretin Is Associated with Earlier Onset of Cardiac Amyloidosis in the African American Population in the Seventh Decade of Life. J. Mol. Diagn. JMD.

[22] Jacobson D.R., Gorevic P.D., Buxbaum J.N. (1990). A Homozygous Transthyretin Variant Associated with Senile Systemic Amyloidosis: Evidence for a Late-Onset Disease of Genetic Etiology. Am. J. Hum. Genet..

[23] Hamour I.M., Lachmann H.J., Goodman H.J.B., Petrou M., Burke M.M., Hawkins P.N., Banner N.R. (2008). Heart Transplantation for Homozygous Familial Transthyretin (TTR) V122I Cardiac Amyloidosis. Am. J. Transplant. Off. J. Am. Soc. Transplant. Am. Soc. Transpl. Surg..

[24] Lopes L.R., Futema M., Akhtar M.M., Lorenzini M., Pittman A., Syrris P., Elliott P.M. (2019). Prevalence of TTR Variants Detected by Whole-Exome Sequencing in Hypertrophic Cardiomyopathy. Amyloid Int. J. Exp. Clin. Investig. Off. J. Int. Soc. Amyloidosis.

[25] Holmgren G., Lundgren E., Kangawa K., Kurihara T., Matsukura S., Matsukura H., Nakazato M., Steen L. (1993). Diagnostic Radioimmunoassay and DNA-Analysis in Swedish and Japanese Patients with Familial Amyloidotic Polyneuropathy. Homozygosity for the TTR Met30 Gene. Acta Neurol. Scand..

[26] Zhao G., Li Z., Araki K., Haruna K., Yamaguchi K., Araki M., Takeya M., Ando Y., Yamamura K. (2008). Inconsistency between Hepatic Expression and Serum Concentration of Transthyretin in Mice Humanized at the Transthyretin Locus. Genes Cells Devoted Mol. Cell. Mech..

[27] Shawky R.M. (2014). Reduced Penetrance in Human Inherited Disease. Egypt. J. Med. Hum. Genet..

[28] Gillmore J.D., Maurer M.S., Falk R.H., Merlini G., Damy T., Dispenzieri A., Wechalekar A.D., Berk J.L., Quarta C.C., Grogan M. (2016). Nonbiopsy Diagnosis of Cardiac Transthyretin Amyloidosis. Circulation.

[29] Garcia-Pavia P., Rapezzi C., Adler Y., Arad M., Basso C., Brucato A., Burazor I., Caforio A.L.P., Damy T., Eriksson U. (2021). Diagnosis and Treatment of Cardiac Amyloidosis: A Position Statement of the ESC Working Group on Myocardial and Pericardial Diseases. Eur. Heart J..

[30] Pilebro B., Suhr O.B., Näslund U., Westermark P., Lindqvist P., Sundström T. (2016). (99m)Tc-DPD Uptake Reflects Amyloid Fibril Composition in Hereditary Transthyretin Amyloidosis. Ups. J. Med. Sci..

[31] Bergström J., Gustavsson A., Hellman U., Sletten K., Murphy C.L., Weiss D.T., Solomon A., Olofsson B.-O., Westermark P. (2005). Amyloid Deposits in Transthyretin-Derived Amyloidosis: Cleaved Transthyretin Is Associated with Distinct Amyloid Morphology. J. Pathol..

[32] Ihse E., Ybo A., Suhr O., Lindqvist P., Backman C., Westermark P. (2008). Amyloid Fibril Composition Is Related to the Phenotype of Hereditary Transthyretin V30M Amyloidosis. J. Pathol..

[33] Musumeci M.B., Cappelli F., Russo D., Tini G., Canepa M., Milandri A., Bonfiglioli R., Di Bella G., My F., Luigetti M. (2020). Low Sensitivity of Bone Scintigraphy in Detecting Phe64Leu Mutation-Related Transthyretin Cardiac Amyloidosis. JACC Cardiovasc. Imaging.

[34] Dasari S., Theis J.D., Vrana J.A., Rech K.L., Dao L.N., Howard M.T., Dispenzieri A., Gertz M.A., Hasadsri L., Highsmith W.E. (2020). Amyloid Typing by Mass Spectrometry in Clinical Practice: A Comprehensive Review of 16,175 Samples. Mayo Clin. Proc..

